# Clinical outcomes of early gastric cardiac cancer treated with endoscopic submucosal dissection in patients with different indications

**DOI:** 10.1186/s12876-021-01700-0

**Published:** 2021-03-12

**Authors:** Ting Fan, Qi Sun, Shouli Cao, Xiangshan Fan, Qin Huang, Shu Zhang, Ying Lv, Xiaoqi Zhang, Tingsheng Ling, Lei Wang, Xiaoping Zou, Guifang Xu

**Affiliations:** 1 Department of Gastroenterology, Nanjing Drum Tower Hospital Clinical College of Nanjing Medical University, Nanjing, China; 2 Department of Pathology, Nanjing Drum Tower Hospital, The Affiliated Hospital of Nanjing University Medical School, Nanjing, China; 3 Department of Gastroenterology, Nanjing Drum Tower Hospital, The Affiliated Hospital of Nanjing University Medical School, Nanjing, China; 4 Department of Pathology and Laboratory Medicine, VA Boston Healthcare System and Harvard Medical School, Boston, USA

**Keywords:** Early gastric cardiac cancer, Endoscopic submucosal dissection, Treatment outcome, Beyond the expanded indication

## Abstract

**Background:**

Endoscopic submucosal dissection (ESD) has been accepted as a standard treatment for early gastric cardiac cancer (EGCC). Here, we investigate the clinical outcomes of the EGCC patients who underwent ESD in different indications.

**Methods:**

From January 2011 to October 2019, we enrolled 502 EGCC lesions from 495 patients which were resected by ESD at our center. We retrospectively analyzed the short-term and long-term clinical outcomes among different indication groups.

**Results:**

The number of the patients in the absolute indication (AI), expanded indication (EI) and beyond the expanded indication (BEI) groups was 265, 137 and 93, respectively. The *en bloc* resection rate was 100%, 100% and 98.9% (*P* = 0.185). The complete resection rate was 99.3%, 98.5% and 74.5%, respectively (*P* < 0.001). During a median follow-up of 48.1 months, the lymph node metastasis rate was 0%, 0% and 2.3% (*P* < 0.001). The distant metastasis rate was 0.4%, 0% and 2.3% (*P* = 0.150). The five-year disease-specific survival rate in the BEI group was 96.6% (*P* = 0.016), compared to 99.6% in the AI group and 100% in the EI group.

**Conclusion:**

The efficacy for ESD patients in EI group was almost equal to the AI group. Patients in the BEI group showed generally favorable clinical outcomes and needed to be carefully checked after ESD. ESD may be an optional treatment for patients unsuitable for gastrectomy.

**Supplementary Information:**

The online version contains supplementary material available at 10.1186/s12876-021-01700-0.

## Background

Gastric cardiac cancer locates in the gastric cardia beneath the gastroesophageal junction [1]. It may cross the gastroesophageal junction to invade the distal esophagus. Our previous study showed 23.3% of early gastric cancer (EGC) was gastric cardiac carcinoma in China [2], which is much higher compared to 7.0% in Japan [3] and 11.9% in western cohort [4]. The incidence of gastric cardiac cancer is rising worldwide for unknown reasons. Gastric cancer is the second most common cause of cancer-related death worldwide [5]. The five-year survival rate was less than 10% in the advanced stage [6]. As a result, it is urgent to perform early detection of gastric cancer through early screening [7]. Endoscopic resection should be applied for EGC [8]. Endoscopic mucosal resection (EMR) and endoscopic submucosal dissection (ESD) exhibited terrific clinical effects in the aspects of complete resection rate and survival outcomes if only the EGC lesions fit the standard and expanded indications [9]. Only a few studies have been published on the exact prevalence of early gastric cardiac cancer (EGCC) due to the rarity and a lack of widely accepted definition for gastric cardiac caner. According to our team’s previous research, compared to distal gastric cancer, early proximal gastric cancers feature smaller size, deeper invasion [1]. Besides, EGCC had a significantly lower risk than non-EGCC, therefore, EGCC may be more suitable for endoscopic resection [1, 2, 10, 11]. Our team have found based on the independent risk factors for gastric cardiac cancer in Chinese patients differ from those of distal gastric cancer or esophageal adenocarcinoma, the classification of gastric cardiac cancer should be considered as a separate gastric cancer entity in the Chinese population [12]. As a consequence, we wonder whether EGCC could achieve a favorable clinical outcome compared to the total EGC. It is widely accepted the EGCC patients of the AI and EI group can obtain excellent treatment outcome. However, a part of specimen is confirmed with lymphovascular invasion, massive submucosal invasion, undifferentiated histology or ulceration after ESD, which lead to “beyond the expanded indication” (BEI).

Until now, the long-term clinical consequence of the BEI group in EGCC patients has not been confirmed. We hope to evaluate the short-term and long-term treatment outcome in all EGCC patients, including the clinical application of the BEI patients.

## Methods

### Patients

From January 2011 to October 2019, 570 patients who underwent endoscopic resection for EGCC at the Affiliated Nanjing Drum Tower Hospital of Nanjing University Medical School were retrospectively enrolled consecutively. EGCC is defined as early cancers with epicenters located in a narrow region of about 3 cm below the esophagogastric junction (EGJ) [13]. EGJ is defined as the distally esophageal palisading vessels. Even if the EGJ was involved, it would be staged using the stomach cancer TNM and stage groups [14]. The exclusion criteria for entry into this study were: (1) low grade epithelial neoplasia (n = 25); (2) other type of carcinoma (n = 10); (3) no carcinoma (n = 28); (4) multiple lesions in different indications (n = 4); (5) insufficient data (n = 8). 495 patients with 502 lesions were enrolled at last. A detailed flow chart on patient selection was exhibited in Additional file 1: Figure 1.

### Endoscopic resection

Each patient underwent a routine gastroscopy to evaluate the status of margin and invasion depth of the EGCC lesion. Abdominal enhanced CT and endoscopic ultrasonography (EUS) were used to determine pretreatment tumor staging. All patients were intravenously anesthetized with midazolam and propofol before endoscopic resection. The ESD procedure was performed by 5 experienced endoscopists at our center with a standard procedure. The standard procedure is as follows: demarcation by indigo-carmine chromoendoscopy and NBI magnifying endoscopy; marking around the lesion; a circular mucosal incision around the marking spots and submucosal resection using a Dual knife step by step. Freshly endoscopically excised specimens were nailed on a dental wax plate, pictured, routinely measured, fixed overnight in 10% neutral formalin buffer then. Next, the specimens were cut evenly into 2-mm intervals. Size of the lesions, depth of tumor invasion, tumor differentiation, lymphovascular invasion and ESD resection margin were examined (Additional file 1: Figure 2).

### Definition

EGCC patients were divided into absolute, expanded, beyond the expanded indication group according to the classification of the Japanese Gastric Cancer Association [9]. Absolute indication was defined as differentiated mucosal cancer smaller than 2 cm in diameter without ulcer. Expanded indications cover one of the circumstances: (1). Differentiated mucosal adenocarcinoma larger than 2 cm without ulceration or lymphovascular invasion; (2). Differentiated ulcer-positive mucosal cancer smaller than 3 cm in size without lymphovascular invasion; (3). Poorly differentiated or undifferentiated ulcer-negative mucosal tumor less than 2 cm in size without lymphovascular invasion; (4). Differentiated ulcer-negative cancer with submucosal invasion less than 500 μm in depth smaller than 3 cm without lymphovascular invasion. When it does not meet absolute and expand criteria, it is considered to be beyond the expanded indication [8].

*En bloc* resection referred to continuous monolithic resection after endoscopic resection. Complete resection was defined as endoscopic *en bloc* resection without histopathological evidence of tumor involvement of margins. Curative resection referred to *en bloc* resection, tumor size less than 2 cm, histologically of differentiated type, pT1a, no residual tumor at the margin and no lymphovascular invasion. For lesions under expanded indication, the resection is considered as curative when all of the following condition are fulfilled: en bloc resection, negative horizontal and vertical margin and no lymphovascular infiltration [8].

The ESD-related complications included significant bleeding, perforation, and stenosis. Significant bleeding was a drop of over 2 g/dL hemoglobin or clinical features instance of endoscopic visible melena or hematemesis. According to whether significant bleeding occurred over 48 h after ESD, we divided it into early and late delayed bleeding [15]. When we observed a hole in the stomach wall under endoscope or a chest X-ray that found free air of the abdomen, it could be diagnosed as a perforation. When the endoscope couldn’t reach the cardiac through distal esophagus because of the excessive contraction of the cardiac, we considered that a stenosis had occurred [16].

Local recurrence was diagnosed once a new tumor was discovered at the prior ESD site. Tumors that were detected at other sites within 1 year after the ESD were interpreted as synchronous tumors, while tumors that were revealed at other sites more than 1 year after the ESD procedure were termed metachronous tumors.

### eCura system

We evaluated the lymph node metastasis rate of EGCC patients after ESD by eCura system. The eCura system consists of five clinicopathological factors, which are scored as followed: 1 point each for tumor size > 30 mm, positive vertical margin, venous invasion, and submucosal invasion over 500 µm and three points for lymphatic invasion. And the patients are divided into three groups according the total points: low risk (0–1 points), intermediate risk (2–4 points) and high risk (5–7 points) [17]. In this study, we used the eCura system to access the disease specific survival and cancer recurrence rate between the two BEI patients with or without additional surgery.

### Follow-up

Patients were required scheduled endoscopy examination in the first 3, 6, 12 months after ESD procedure, and once a year thereafter. The surveillance of thoracic and abdominal CT was performed after 6, 12 months. When endoscopists found a new tumor at a previous ESD location, local recurrence was considered to occur. Synchronized tumors were diagnosed once tumors were found at other sites within a year of ESD, and metachronous tumors were that occurred at the other sites over a year after ESD.

### Statistical analysis

Statistics was analyzed by SPSS 23.0 (IBM, Armonk, New York, USA). Student’s tor Chi-square test was applied to assess the differences between measurement data. Chi-square test or Fisher’s exact test was called for the analyses of categorical variables to obtain statistical significance. Kaplan–Meier method was used to estimate the survival rates. A *P* value of < 0.05 was regarded as statistically significant.

## Results

### Baseline demographic and clinical features

A total of 570 patients had undergone ESD and 495 patients with 502 lesions were enrolled with the following criteria, among whom 265 patients with 271 lesions were enrolled in the AI group, 137 patients with 137 lesions in the EI group and 93 patients with 94 lesions in the BEI group. 5 patients were withdrawn during the follow-up. 450 patients were enrolled for the long-term outcome analysis in the end (Additional file 1: Figure 3).

As shown in Table 1, the average age was 65.5 (range 44–87) and 82% were male. 291 patients (58.8%) had a smoking history, 263 patients (53.1%) had a drinking habit and 230 patients (46.5%) regularly ate pickled food. Nearly half of the patients (40.8%) had a family tumor history. The most common complication was hypertension (32.7%, 162/495). 14 patients (2.8%) suffered from gastroesophageal reflux disease.

**Table 1:** Demographic and clinical features characteristics of early gastric cardiac cancer

Characters	Total (n = 495)	Absolute indication (n = 265)	Expanded indication (n = 137)	Beyond expanded indication (n = 93)	*P* value
Gender, number (%)					0.093
Male	406 (82.0)	214 (80.8)	120 (87.6)	72 (77.4)	
Female	89 (18.0)	51 (19.2)	17 (12.4)	21 (22.6)	
Age (year)	65.5 (44–87)	65.1 (45–85)	65.4 (45–79)	67.2 (44–87)	0.059
Smoke, number (%)	291 (58.8)	152 (57.4)	89 (65.0)	50 (53.8)	0.187
Drink, number (%)	263 (53.1)	137 (51.7)	79 (57.7)	47 (50.5)	0.449
Pickled food, number (%)	230 (46.5)	113 (42.6)	74 (54.0)	43 (46.2)	0.095
BMI (kg/m^2^) (SD)	23.0 (3.2)	23.1 (3.4)	22.9 (3.1)	23.2 (3.0)	0.851
Family history, number (%)	202 (40.8)	93 (35.1)	63 (46.0)	46 (49.5)	0.018
Complications, number (%)					0.767
Diabetes mellitus	36 (7.3)	14 (5.3)	13 (9.5)	9 (9.7)	
Hypertension	162 (32.7)	89 (33.6)	40 (29.2)	33 (35.5)	
Hyperlipidemia	9 (1.8)	6 (2.3)	1 (0.7)	2 (2.2)	
Cardiovascular disease	20 (4.0)	12 (4.5)	4 (2.9)	4 (4.3)	
Hepatitis B	25 (5.1)	14 (5.3)	8 (5.8)	3 (3.2)	
Chromic pulmonary disease	32 (6.5)	17 (6.4)	10 (7.3)	5 (5.4)	
Reflux esophagitis	14 (2.8)	6 (2.3)	3 (2.2)	5 (5.4)	

### Endoscopic and pathological characteristics

The average tumor size was 12 mm (range 2–20) in the AI group, 23 mm (range 6–66) in the EI group, and 27 mm (range 5–65) in the BEI group (*P* < 0.001) (Table 2). Most lesions were smaller than 20 mm (73.7%, 370/502). The most common site of EGCC was posterior curvature (51.0%, 256/502), followed by lesser curvature (42.2%, 212/502). The most common endoscopic infiltration growth pattern was INFa (66.3%, 333/502). The most common macroscopic pattern, in the descending order, was 0-IIc (45.2%, 227/502), 0-IIa + IIc (27.9%, 140/502), 0-IIa (14.9%, 75/502), 0-IIb (10.0%, 50/502), 0-III (1.2%, 6/502), and 0-I (0.8%, 4/502). All tumor lesions in the AI group were intramucosal and differentiated. Instead, in the BEI group, 5 patients (5.3%, 5/94) had undifferentiated carcinoma and the majority (94.6%, 89/94) had submucosal invasion (*P* < 0.001). 24 patients (4.8%, 24/502) had ulceration over the three group and 13 patients (13.8%, 13/94) in the BEI group were positive. Focal distal esophageal involvement was detected in 12.0% (60/502) in all patients. Lymphovascular invasion was identified in 3.0% (15/502) in all patients. Gastritis cystica profunda was found in over 24% of lesions (122/502) and there were no differences among the three groups. Overall positive rate of *helicobacter pylori* was 56.8% and the difference among the three groups was non-significant. Atrophic gastric carditis was found in 89.4% (449/502).

**Table 2:** Endoscopic and Pathological Characteristics of early gastric cardiac cancer

Characters	Total (n = 502)	Absolute indication (n = 271)	Expanded indication (n = 137)	Beyond expanded indication (n = 94)	*p* value
Direction, number (%)					0.151
Anterior wall	13 (2.6)	9 (3.3)	2 (1.5)	2 (2.1)	
Posterior wall	256 (51.0)	147 (54.2)	68 (49.6)	41 (43.6)	
Lesser curvature	212 (42.2)	100 (36.9)	64 (46.7)	48 (51.1)	
Greater curvature	15 (3.0)	12 (4.4)	2 (1.5)	1 (1.1)	
Circumferential	6 (1.2)	3 (1.1)	1 (0.7)	2 (2.1)	
Mean Tumor size (mm), number (%)					< 0.001
≤ 20	370 (73.7)	271 (100)	52 (38.0)	47 (50.0)	
20–30	87 (17.3)	0 (0)	67 (48.9)	20 (21.3)	
> 30	45 (9.0)	0 (0)	18 (13.1)	27 (28.7)	
Invasion depth, number (%)					< 0.001
LPM	168 (33.5)	137 (50.6)	29 (21.2)	2 (2.1)	
MM	189 (37.6)	134 (49.4)	52 (38.0)	3 (3.2)	
SM1	74 (14.7)	0 (0)	56 (40.9)	18 (19.1)	
SM2	71 (14.1)	0 (0)	0 (0)	71 (75.5)	
Endoscopic infiltration (INF) model, number (%)					< 0.001
INFa	333 (66.3)	198 (73.1)	88 (64.2)	47 (50.0)	
INFb	143 (28.5)	64 (23.6)	44 (32.1)	35 (37.2)	
INFc	26 (5.2)	9 (3.3)	5 (3.6)	12 (12.8)	
Macroscopic type, number (%)					< 0.001
0-I	4 (0.8)	3 (1.1)	0 (0)	1 (1.1)	
0-IIA	75 (14.9)	49 (18.1)	12 (8.8)	15 (3.0)	
0-IIB	50 (10.0)	32 (11.8)	16 (11.7)	1 (1.1)	
0-IIC	227 (45.2)	112 (41.3)	74 (54.0)	41 (43.6)	
0-IIA + IIC	140 (27.9)	75 (27.7)	32 (23.4)	33 (35.1)	
0-III	6 (1.2)	0 (0)	3 (2.2)	3 (3.2)	
Ulceration, number (%)					< 0.001
Presence	24 (4.8)	0 (0)	11 (8.0)	13 (13.8)	
Absence	478 (95.2)	271 (0)	126 (92.0)	81 (86.2)	
Pathological type, number (%)					< 0.001
WD	279 (55.6)	179 (66.1)	78 (56.9)	22 (23.4)	
MD	123 (24.5)	51 (18.8)	31 (22.6)	41 (43.6)	
WD + MD	92 (18.3)	41 (15.1)	25 (18.2)	26 (27.7)	
PD	8 (1.6)	0 (0)	3 (2.2)	5 (5.3)	
Lymphatic and vascular invasion, number (%)					< 0.001
Presence	15 (3.0)	0 (0)	1 (0.7)	14 (14.9)	
Absence	487 (97.0)	271 (100)	136 (99.3)	80 (85.1)	
Esophagus involvement, number (%)					< 0.001
Presence	60 (12.0)	25 (9.2)	12 (8.8)	23 (24.5)	
Absence	462 (88.0)	246 (90.8)	125 (91.2)	71 (75.5)	
Gastritis cystica profunda, number (%)					0.062
Presence	122 (24.3)	55 (20.3)	40 (29.2)	27 (28.7)	
Absence	380 (75.7)	216 (79.7)	97 (70.8)	64 (71.3)	
HP infection, number (%)					0.256
Presence	285 (56.8)	149 (55.0)	87 (63.5)	54 (57.4)	
Absence	217 (43.2)	122 (45.0)	50 (36.5)	40 (42.6)	
Atrophic gastritis, number (%)					0.864
Presence	449 (89.4)	243 (89.7)	121 (88.3)	85 (90.4)	
Absence	53 (10.6)	28 (10.3)	16 (11.7)	9 (9.6)	

### Short-term outcomes and complications based on indication

As Table 3 clarified, the overall *en bloc* resection rate was 99.8% (501/502), among which only one lesion in the BEI group was broken. Complete resection rate of all cases was 94.4% (474/502); 99.3% (269/271), 98.5% (135/137) and 74.5% (70/94), respectively (*P* < 0.001). The rate of curative resection was 79.9% in all the patients: 98.5% (267/271), 97.8% (134/137) and 0% (0/94), respectively (*P* < 0.001). Factors associated non-curative resection included lymphovascular infiltration (n = 15), submucosal invasion (n = 71), positive vertical margin (n = 4), positive lateral margin (n = 21), or positive lateral and vertical margin (n = 3).

**Table 3:** Short-term outcomes and complications based on indication

Characters	Total (n = 502)	Absolute indication (n = 271)	Expanded indication (n = 137)	Beyond Expanded indication (n = 94)	*p* value
*En bloc* resection, number (%)	501 (99.8)	271 (100)	137 (100)	93 (98.9)	0.185
Complete resection, number (%)	474 (94.4)	269 (99.3)	135 (98.5)	70 (74.5)	< 0.001
Curative resection, number (%)	401 (79.9)	267 (98.5)	134 (97.8)	0 (0)	< 0.001
Free vertical margin, number (%)	495 (98.6)	271 (100)	136 (99.3)	88 (93.6)	< 0.001
Free lateral margin, number (%)	478 (95.2)	269 (99.3)	135 (98.5)	74 (78.7)	< 0.001
Complication, number (%)					
Significant bleeding	12 (2.4)	5 (1.8)	2 (1.5)	5 (5.3)	0.128
Early delayed	8 (1.6)	1 (0.4)	2 (1.5)	5 (5.3)	0.004
Late delayed	4 (0.8)	4 (1.5)	0 (0)	0 (0)	0.306
Perforation	1 (0.2)	1 (0.4)	0 (0)	0 (0)	1
Stenosis	18 (3.6)	4 (1.5)	10 (7.3)	4 (4.3)	< 0.001
Median procedure time (min) (range)	65.3 (10–353)	59.9 (10–353)	70.8 (18–223)	72.9 (10–246)	0.007
Median hospital stays (day) (range)	6.6 (2–19)	6.4 (3–14)	7.0 (4–19)	6.8 (2–14)	0.012

In the aspect of complications, 12 cases had significant bleeding, in which 8 cases were early delayed bleeding, 4 cases were late delayed bleeding. The early delayed bleeding rate was 0.4% (1/271), 1.5% (2/137), 5.3% (5/94) in the AI, EI and BEI group, respectively, which was a difference of statistics among the three groups (*P* = 0.004). This could be due to the larger size of tumor lesions and submucosal invasion. Luckily, all the patients who suffered from bleeding were successfully managed by endoscopic hemostasis (n = 11) and the use of hemostatic drugs such as octreotide acetate and somatostatin (n = 1). Perforation was found in 1 AI patient and this patient's symptoms improved after conservative treatment. Stenosis was a common complication of patients who underwent ESD and was found in 3.6% (18/502) patients, among whom 4 patients (1.5%, 4/271) were in the AI group, 10 patients (7.3%, 10/137) belonged to the EI group and 4 patients (4.4%, 4/94) were members of the BEI group (*P* = 0.002). After balloon dilation, the symptoms of stenosis were easily improved. The presence of stenosis was positively related to a circumferential extent of the mucosal defect of > 3/4 or longitudinal extent of > 5 cm in length according to the previous investigation [18]. The median time of ESD operations was 65.3 min (range 10–246), while the BEI patients required longer procedure time, which was 72.9 min (range 18–223) (*P* = 0.007). The average hospitalization time was 6.6 days (range 2–19). Similarly, the EI and BEI patients stayed longer than the AI patients (*P* = 0.012).

### Long-term therapeutic outcomes according to the indication

**Fig.1:**
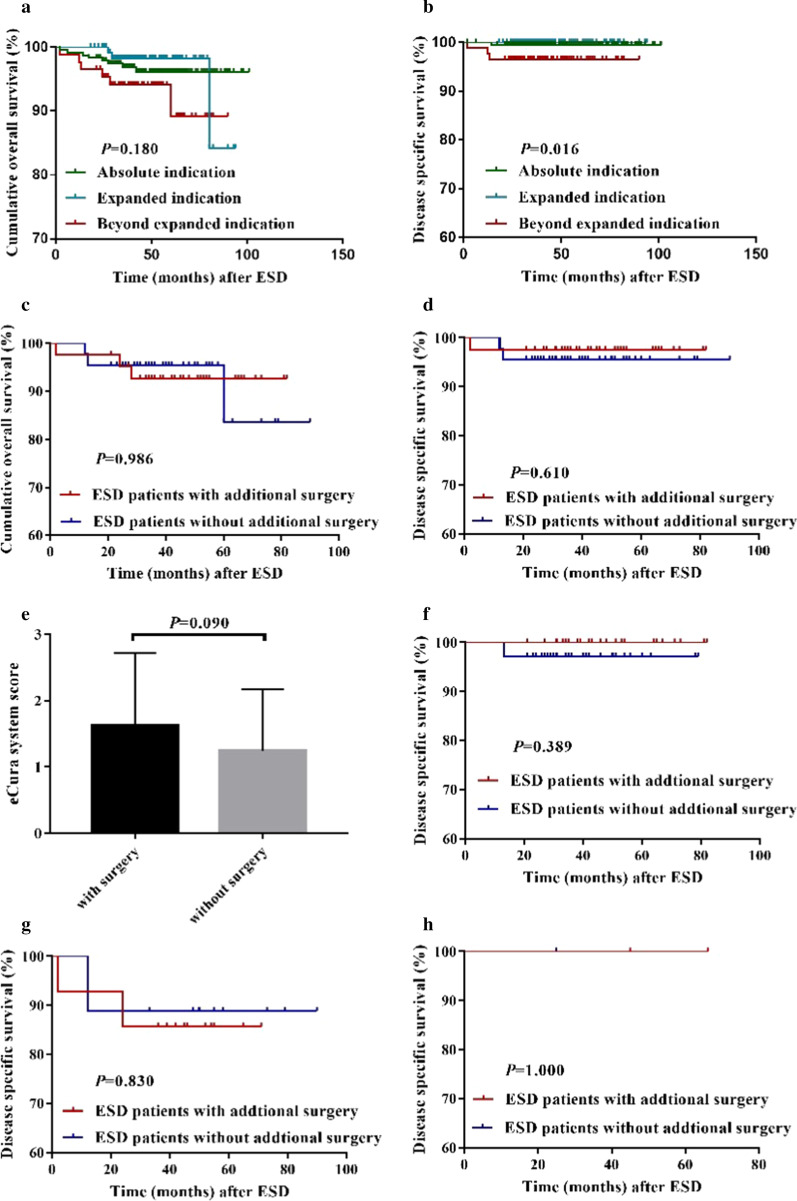
Kaplan–Meier plots of survival among patients after ESD. **a** Overall survival between three groups. **b** Disease specific survival between three groups. **c** Overall survival between BEI patients with or without additional surgery. **d** Disease specific survival between BEI patients with or without additional surgery. **e** The eCura system score of the BEI patients with or without additional surgery. **f** The disease specific survival of the BEI patients with or without additional surgery in low risk category. **g** The disease specific survival of the BEI patients with or without additional surgery in intermediate risk category. **h** The disease specific survival of the BEI patients with or without additional surgery in high risk category

During the median follow-up of 48.1 months (range 18–101), 45 patients (9.1%, 45/495) were lost during follow-up. Additional surgery was carried out for 7 AI patients (2.9%, 7/239), 10 EI patients (8.1%, 10/124) and 42 BEI patients (48.3%, 42/87) (Table 4). Local recurrence was found in only one AI patient because of the residual tumor (*P* = 1.000). Synchronous cancer was detected in 39 patients (8.7%, 39/450), and there was no statistically significant difference among the three groups. The similar conclusion was achieved for metachronous cancer. During the follow-up, 2 BEI patients (2.3%, 2/87) were found to have lymph node metastasis (*P* = 0.037). These patients underwent the additional surgery and survived well during long-term follow-up. Distant metastasis was developed in one AI patient and two BEI patients (Table 4). They didn’t receive the additional surgery and passes away unfortunately. Disease-specific death occurred in 4 patients (0.9%, 4/450); 1 (0.4%, 1/239) in the AI group, no patient in the EI group and 3 (3.4%, 3/87) in the BEI group (*P* = 0.030). The AI patient was dead because of metastasis whose lateral margin was positive and he refused the additional surgery. One BEI patient was dead due to the significant bleeding after the additional surgery. The other two patients passed away related to distant metastasis without additional surgery (Table 5). Five-year overall survival rates were 96.1% (AI), 98.3% (EI) and 89.1% (BEI), which didn’t have any statistic difference (*P* = 0.180) (Fig. 1a). Five-year disease-specific survival rates of three groups were 99.6%, 100% and 96.6% in the AI, EI and BEI group, respectively, which was significantly different between each other (*P* = 0.016) (Fig. 1b). Besides, we analyzed whether the additional surgery was necessary for the patients in the BEI group. After ESD surgery, 93 patients were enrolled into the BEI group based on postoperative pathology. 6 patients were lost during the follow-up. 42 of the remaining 87 BEI patients underwent additional surgery according to the doctor's advice. As we can see in Fig. 1c, d, the additional surgery didn’t have an influence on neither the overall survival nor the disease specific survival.

**Table 4:** Long-term therapeutic outcomes according to EGCC subgroup

Characters, n (%)	Total (n = 450)	Absolute indication (n = 239)	Expanded indication (n = 124)	Beyond expanded indication (n = 87)	*p* value
Median time, months (SD)	48.1 (18.8)	49.2 (20.0)	47.5 (17.1)	45.7 (17.5)	0.291
Local recurrence, number (%)	1 (0.2)	1 (0.4)	0 (0)	0 (0)	1.000
Synchronous cancer, number (%)	39 (8.7)	26 (10.9)	7 (5.6)	6 (6.9)	0.197
Metachronous cancer, number (%)	7 (1.4)	6 (2.5)	1 (0.8)	0 (0)	0.340
Additional surgery, number (%)	59 (13.1)	7 (2.9)	10 (8.1)	42 (48.3)	< 0.001
Observation, number (%)	391 (86.9)	232 (97.1)	114 (91.9)	45 (51.7)	< 0.001
Lymph node metastasis, number (%)	2 (0.4)	0 (0)	0 (0)	2 (2.3)	0.037
Distant metastasis, number (%)	3 (0.7)	1 (0.4)	0 (0)	2 (2.3)	0.150
Overall death, number (%)	17 (3.8)	8 (3.3)	3 (2.4)	6 (6.9)	0.212
Disease related death, number (%)	4 (0.9)	1 (0.4)	0 (0)	3 (3.4)	0.030

**Table 5:** Clinical characteristics of patients with disease related death after endoscopic submucosal dissection

No	Age (year)	Gender	Tumor size (mm)	Lateral margin	Vertical margin	Lympho-vascular invasion	Pathological type	Invasion depth	Ulcer	Additional surgery	Group	Death reason
1	60–70	M	30	N	N	N	WD + MD	SM2	N	Y	BEI	Bleeding
2	70–80	M	12	N	N	Y	MD	SM2	N	N	BEI	Metastasis
3	40–50	F	5	Y	N	N	WD	SM2	N	N	BEI	Metastasis
4	70–80	M	9	Y	N	N	WD	M3	N	N	AI	Metastasis

We wondered why the disease specific survival was no significantly difference between the two BEI group with or without the additional surgery. So, we applied the eCura system to evaluate whether there was any difference between these two groups. According to the generally accepted method, we divided the BEI patients with or without additional surgery into three categories (Additional file 1: Table 1). The detailed scores had no difference between the two groups (Fig. 1e), which meant the LNM risk was resemble between the BEI patients with or without surgery, consistent with the survival analysis. In low risk category, disease specific survival was higher in the patient accepted the additional surgery (100% vs 97.1%), but was no significant difference for the two groups (*p* = 0.389; Fig. 1f). In the intermediate risk category, one patient in each of the two groups had a disease-related death. We could achieve the similar conclusion: the patients received the additional surgery has higher five-year disease-free survival rate, but there was no significantly different (92.9% vs 88.9%, *p* = 0.830; Fig. 1g). Since there was no disease-related death of the patients in the high-risk category and a small number of cases, we could not achieve a reliable survival analysis for the data (Fig. 1h). In summary, the patients in the BEI group may not benefit from the additional surgery after ESD in our research due to the small account of data.

## Discussion

This is a large-scale research to compare the short- and long-term clinical outcomes among the absolute indication, expanded indication and beyond the expanded indication EGCC patients of endoscopic resection. Across hospitals all over the world, endoscopic resection has gain widely acceptation as a standard treatment of EGC under absolute and expanded criteria as an alternative to surgical resection of distal esophagus and proximal stomach [19–23]. ESD has advantages in lower rates of acute complications and comparable overall survival [22]. Several studies have displayed the effectiveness and safety of endoscopic resection for adenocarcinoma of EGJ [24, 25]. For the cancer located in EGJ, it encompasses both gastric cardia adenocarcinoma (GEA) and Barrett’s esophageal adenocarcinoma (BEA) due to short-segment Barrett’s esophagus. Few researches have specially focused on the clinical outcome of ESD for these gastric cardiac adenocarcinomas. Osumi et al*.* reported curative resection rate was higher in GCA group (81%) than BEA group (60%) [26]. Jang et al. enrolled 82 patients with gastric cardiac tumors and the en bloc resection, complete resection, and curative resection rates were 87%, 79%, and 66% [27]. A handful of researches have proved ESD is an efficient way to cure EGCC patients. Due to the small sample size, more persuasive studies needed to be conducted.

In our study, we enrolled 495 patients with 502 ESD-related EGCC lesions. The effectiveness of ESD was confirmed by comparing the short and long-term clinical consequence among the AI, EI and BEI groups. No significant difference was found in demographic statistics for these three groups. In the present study, patients in BEI group had more family history than AI and EI groups (*P* = 0.018). Increased tumor size, deeper tumor invasion, presence of ulcer and poor differentiated were significantly different among the three groups, which was consistent with the definition. In our research, atrophy of the mucosa around the tumor lesion could be found in the vast majority EGCC patients. Atrophic gastritis and intestinal metaplasia were the most significant risk factors for gastric cancer [28, 29], thus the endoscopic surveillance in these high risk patients were expected to be extremely important.

The complete resection and curative resection rates in the AI group were meaningfully higher in comparison with the other two groups (*P* < 0.001). This was closely related to the positive vertical and lateral cutting margins in the EI and BEI groups. Suzuki et al*.* drew the conclusion through their research that positive margins with submucosal infiltration (odds ratio 3.6) and lymphovascular invasion (odds ratio 3.5) had significant correlation with lymph node metastasis and patients who didn’t meet curative resection especially with lymphovascular invasion or positive margin with submucosal invasion should receive additional gastrectomy [30]. Positive lateral margin was related closely to a mixed-type carcinoma, larger than 3 cm in size and the upper one third of stomach, reported by Fu et al. [31]. However, there is no research about the risk factor of vertical and lateral incomplete resection in EGCC patients which needs to study in depth.

In the AI and EI group, the five-year overall and disease-specific survival rates were no statistic differences with 96.1–98.3% and 99.6–100%. No case in the AI and EI group was found lymph node metastasis until now. Only one AI patient died because of a positive lateral margin. We notified the patient that there was a problem with the pathological result and additional surgery was required. However, the patient had some concerns, thinking that he was old, may have a higher risk of surgery, and have a slower recovery. He did not want to perform a second surgery in a short time, so he refused the additional surgery and died 14 months after ESD unfortunately. These findings were consistent with the previous results and the five-year survival rate of ESD surgery was comparable to that of radical gastrectomy [32–34]. The five-year overall survival rate of mucosal EGC was 99% and the five-year overall survival rate of submucosal EGC was 96% by surgical treatment according to the National Cancer Center of Japan [8], which was consistent with the long-term outcome of EGCC patients after ESD at our center. In short, if only the tumor lesions met the absolute or expanded indication, endoscopic resection could achieve satisfactory safety and efficacy from the long-term follow-up. As a consequence, conclusion could be drawn that endoscopic resection was an effective way for the EGCC patients within the AI and EI group similar to other studies [4, 35, 36].

However, there are still many concerns in terms of the BEI group. In this study, the rate of *en bloc* resection arrived at 98.9% (93/94) in BEI group. Yet, The BEI group showed lower rates of complete resection (74.5%, 70/94) (*P* < 0.001). The reason for the result was mainly related to tumor lesion size, lymphatic/vascular invasion, deep submucosal invasion and undifferentiated histology. These factors were closely related to lymph node metastasis and recurrence [37–39]. As a result, these patients were not suitable for endoscopic resection from the results of previous researches. We evaluated the tumor lesions by EUS, CT or biopsy before ESD and we found some cases were under massive submucosal invasion. Gastrectomy, especially proximal gastrectomy, is the most common surgical treatment of gastric cardiac cancer for the patients unsuitable for ESD. However, some patients suffered from severe postoperative complications, such as anastomotic leakage, reflux esophagitis and anastomotic stricture after proximal gastrectomy. According to the recent reports, the short-term and long-term gastrectomy complications rates could be up to 20% compared with less than 10% by ESD [40, 41]. Based on the EGC guideline, BEI group should be recommended additional surgery strongly, however, in our study, only half BEI patients received additional surgery. These patients were aware of beyond the expanded criteria but they still insisted endoscopic resection when they met advanced age, serious underlying diseases and chose the less invasive strategy instead of the surgery. In addition, 2.4% (1/42) patient dead because of massive hemorrhage after the additional gastrectomy, which was far higher than the mortality rate reported in the literatures. It has been reported that the rate of delayed massive hemorrhage after gastrectomy in patients with gastric cancer was 0.9% with 0.2% death rate [42]. Park’s study showed the incidence of the postoperative bleeding was 0.8% and the subsequent mortality rate was 0.08% [43]. This may be the result of bias due to the low number of cases included in our research. According to our research, the death rate of patients without additional surgery in the BEI group is lower than that of patients with additional surgery, which caught our attention (2.4% vs 4.4%). Due to the small number of cases, there was no significant difference in 5-year survival with or without additional surgery. The content of this part needs further research. Although surgical procedures have certain risks, for these two young BEI patients who died of metastases due to no additional surgery, perhaps additional surgery after ESD may give them the possibility of long-term survival.

We found that the five-year overall survival rate and the disease specific survival rate for the BEI group patients up to 89.1% and 96.6% during the long-term follow-up at our center. In other centers, endoscopists found the similar results which the five-year disease-free survival rate of BEI patients after ESD could be as high as 90% or more, which was quite effective [32, 44, 45]. Yet, the disease-specific survival of the BEI group was still significantly lower than that of the AI and EI groups, which suggests that we need to carefully consider the next step of treatment for these patients in the BEI group after ESD. After we have fulfilled the obligation of the doctor to inform, the patient should follow the doctor’s advice and perform additional surgical operations to prevent the occurrence of tragedies. The rates of R0 resection and curative resection in BEI group may be lower than the patients in the indications, but in the long run, the survival rate in the BEI group patients who underwent ESD instead of radical gastrectomy still showed a favorable performance. According to a large-scale multi-center study conducted by Takizawa and Hatta et al., the authors found that out of 905 non-curative resection patients without additional surgery, a total of 27 patients had recurrence (27/905, 3.0%), of which distant metastasis was the most common way of recurrence (15/27, 60%) [46]. In contrast, in our study, 4.4% of patients with non-curative resection without additional surgery died of distant metastases. It reminds us that endoscopists would strongly recommend the BEI patients to receive additional surgery after ESD. The necessity for additional surgery needs to be repeatedly emphasized to patients to increase their attention. Besides, between the two groups with or without surgery, we concluded that there was no significant statistical difference from the K-M survival curve. Different from the previous studies [40, 47], those patients under BEI group didn’t benefit from the additional surgery neither the five-year overall survival nor the five-year disease-specific survival. Based on the eCura system, it could be a useful aid for selecting the appropriate treatment strategy after the noncurative ESD for EGCC [39]. If we followed up long enough or enrolled more cases, a different conclusion might jump to us.

Our study still has several limitations. First, this research is a single center retrospective cohort analysis which may leads to a section bias and referral bias. Besides, an average of 48.1 months of follow-up may not allow us to find the significant difference in the survival rate of the BEI patients with or without additional surgery. In this rather small subgroup, the survival did not show significant differences possibly because of the minor case number. In addition, we are urge to learn about the risk factors of positive margins and noncurative resection.

## Conclusion

Endoscopic resection of EGCC could achieve a favorable short-term and long-term prognosis for patients with absolute and expanded indication. Patients in beyond the expanded indication showed generally favorable clinical outcomes and needed to be carefully checked after ESD. ESD may be an optional treatment for the patients unsuitable for gastrectomy.

## Supplementary Information

**Additional file 1: Figure 1**. Flow diagram for the patients in this study. **Figure 2**. The endoscopic submucosal dissection for early gastric cardiac cancer. **Figure 3**. Patient treatment flow chart. **Table 1**. Disease specific survival at 3 and 5 years among BEI patients with or without additional surgery after ESD for EGCC which were divided into three risk categories according to the eCura system.

## Data Availability

The datasets used during the current study are available from the corresponding author on reasonable request.

## Abbreviations

EGC: Early gastric cancer
EGCC: Early gastric cardiac cancer
EGJ: Esophagogastric junction
ESD: Endoscopic submucosal dissection
EMR: Endoscopic mucosal resection
AI: Absolute indication
EI: Expanded indication
BEI: Beyond the expanded indication
