# Supplementary material for: Clinical outcomes of early gastric cardiac cancer treated with endoscopic submucosal dissection in patients with different indications
Source: BMC Gastroenterol. 2021 Mar 12;21:119. doi: 10.1186/s12876-021-01700-0 (PMC7953789; doi:10.1186/s12876-021-01700-0)
Supplement: Supplementary file 1 — Additional file 1: Figure 1. Flow diagram for the patients in this study. Figure 2. The endoscopic submucosal dissection for early gastric cardiac cancer. Figure 3. Patient treatment flow chart. Table 1. Disease specific survival at 3 and 5 years among BEI patients with or without additional surgery after ESD for EGCC which were divided into three risk categories according to the eCura system. [file 12876_2021_1700_MOESM1_ESM.docx]

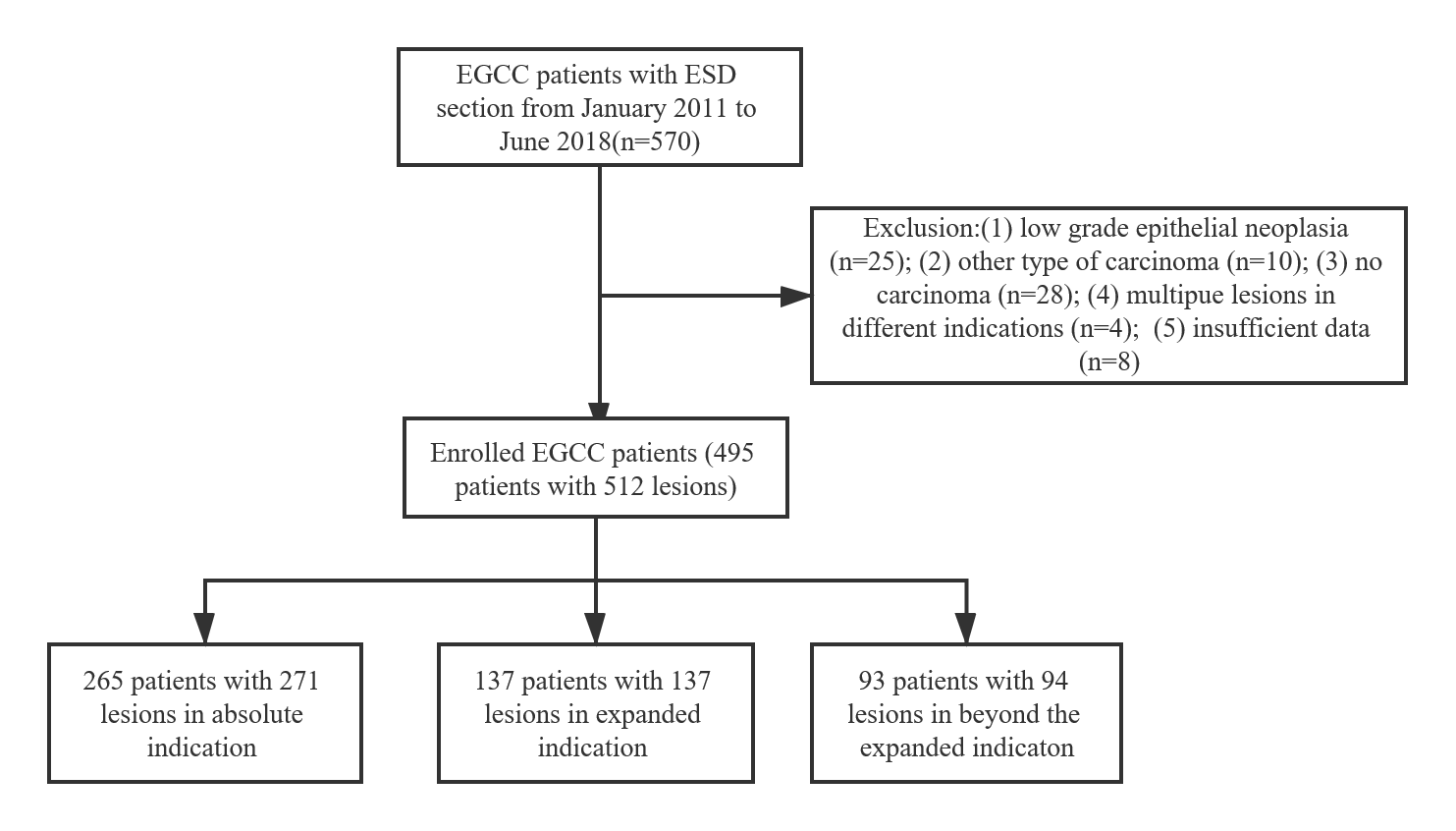


Supplementary Figure 1 Flow diagram for the patients in this study.


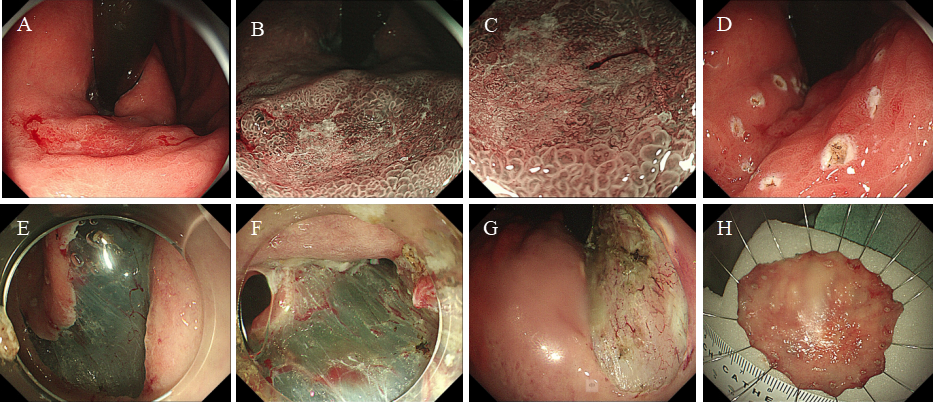


Supplementary Figure 2 The endoscopic submucosal dissection for early gastric cardiac cancer. A: White-light endoscopy (WLE) image of an early gastric cardiac cancer. B: The edge of the tumor lesion in magnifying endoscopy with narrow-banding imaging (ME-NBI). C: The destructive microstructures and fine reticular microvascular morphology in ME-NBI. D: Marking the edge of tumor margin. E: Cut around the marking points. F: Submucosal dissection. G: Wound surface after dissection. H：The resected specimen.


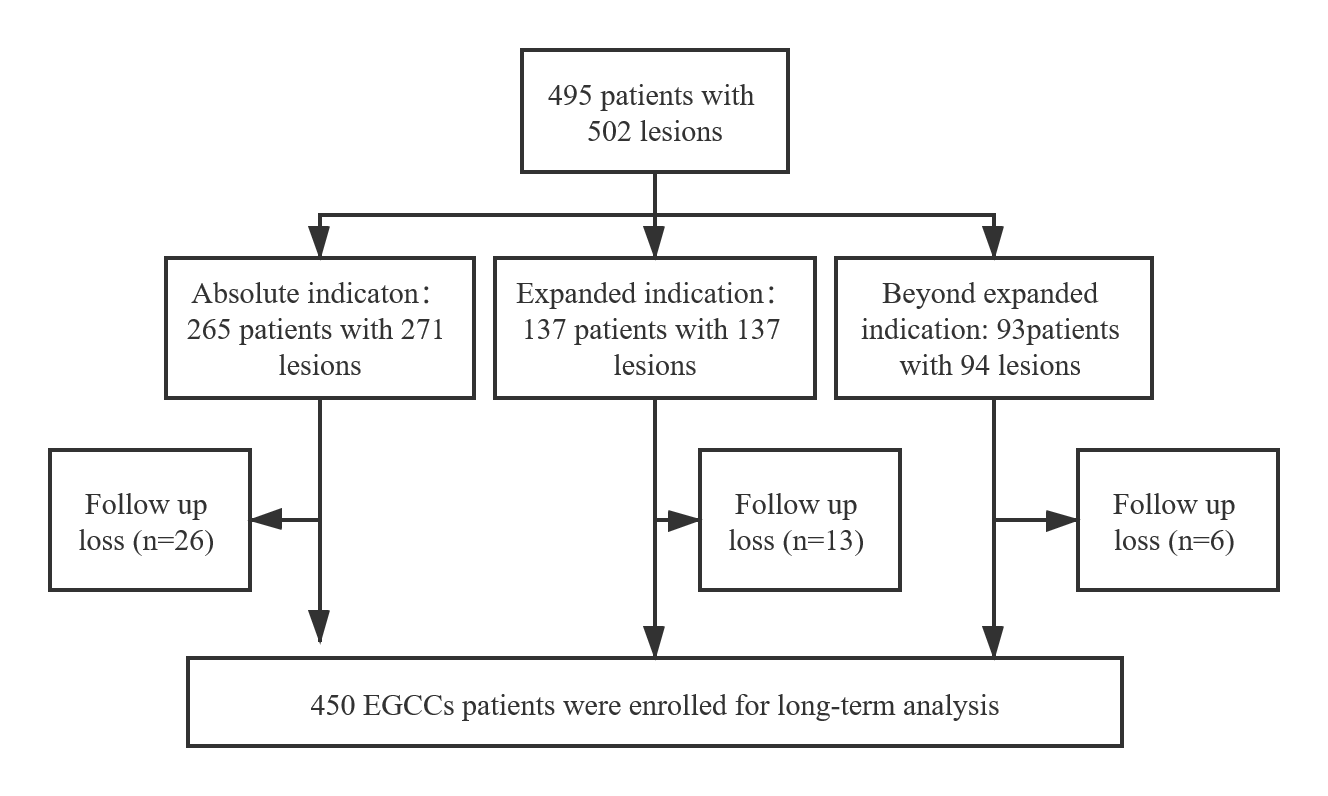


Supplementary Figure 3 Patient treatment flow chart


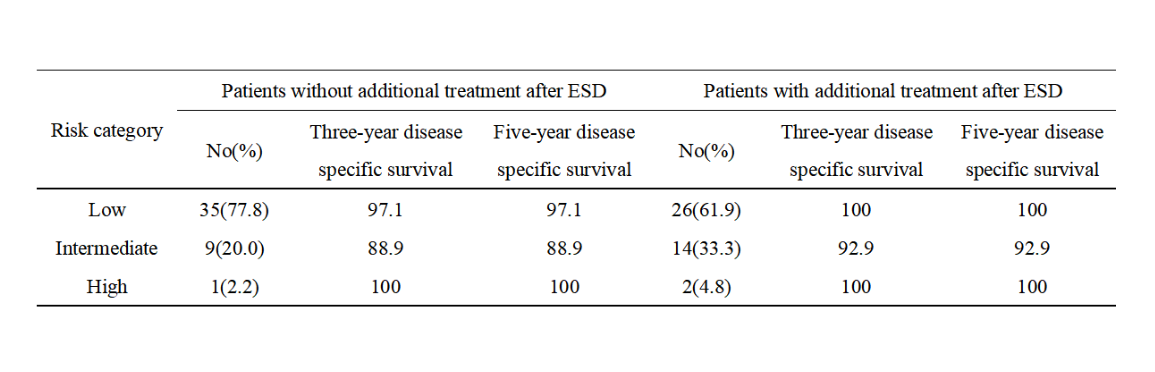


Supplementary table 1 Disease specific survival at 3 and 5 years among BEI patients with or without additional surgery after ESD for EGCC which were divided into three risk categories according to the eCura system
